# Global and local drivers of *Echinococcus multilocularis* infection in the western Balkan region

**DOI:** 10.1038/s41598-023-46632-9

**Published:** 2023-12-01

**Authors:** Sibusiso Moloi, Tamás Tari, Tibor Halász, Bence Gallai, Gábor Nagy, Ágnes Csivincsik

**Affiliations:** 1 One Health Working Group, Institute of Physiology and Animal Nutrition, Kaposvár Campus, Hungarian University of Agriculture and Life Sciences, Guba S. U. 40., Kaposvár, 7400 Hungary; 2 Institute of Wildlife Biology and Management, Faculty of Forestry, University of Sopron, Sopron, 9400 Hungary; 3 Zselic Wildlife Estate, Somogy County Forest Management and Wood Industry Share Co. Ltd., Kaposvár, 7400 Hungary; 4 Institute of Geomatics and Civil Engineering, Faculty of Forestry, University of Sopron, Sopron, 9400 Hungary

**Keywords:** Zoology, Diseases

## Abstract

The cestode*, Echinococcus multilocularis*, is one of the most threatening parasitic challenges in the European Union. Despite the warming climate, the parasite intensively spread in Europe's colder and warmer regions. Little is known about the expansion of *E. multilocularis* in the Balkan region. Ordinary least squares, geographically weighted and multi-scale geographically weighted regressions were used to detect global and local drivers that influenced the prevalence in red foxes and golden jackals in the southwestern part of Hungary. Based on the study of 391 animals, the overall prevalence exceeded 18% (in fox 15.2%, in jackal 21.1%). The regression models revealed that the wetland had a global effect (β = 0.391, *p* = 0.006). In contrast, on the local scale, the mean annual precipitation (β = 0.285, *p* = 0.008) and the precipitation seasonality (β = − 0.211, *p* = 0.014) had statistically significant effects on the infection level. The geospatial models suggested that microclimatic effects might compensate for the disadvantages of a warmer Mediterranean climate. This study calls attention to fine-scale analysis and locally acting environmental factors, which can delay the expected epidemic fade-out. The findings of our study are suggested to consider in surveillance strategies.

## Introduction

The cestode*, Echinococcus multilocularis*, is one of the most threatening parasitic challenges in the European Union^1^. The adult worms can be found dominantly in the small intestines of wild canids, but in some regions of the world, domesticated dogs can also play a central role in its biological cycle^2,3^. Besides the definitive hosts (canine species), mainly rodents can serve as intermediate hosts in the life cycle. Canines release eggs into the environment via faecal material. If an adequate intermediate host ingests eggs, the release of the oncospheres penetrates different organs (mainly the liver) via circulation, where the oncospheres develop numerous multi-chambered, thin-walled cysts with infective protoscolices in them. A definitive host can get infected after consumption of a cyst- and protoscolex-containing intermediate host, which are dominantly arvicolids and muskrats in Europe^2,4^. The most prevalent definitive host is the red fox in Europe, but in the Balkan region, a similar infection level could be observed in the golden jackal^5–8^. This phenomenon was also proved in a southwestern Hungarian study wherein 173 jackals were examined to determine the role of this carnivore in local *E. multilocularis* endemic. The results of the five-years duration study showed the prevalence of the parasite was up to 15%^7^. Humans could be infected accidentally by ingestion of eggs either in direct contact with the definitive host or after eating or drinking contaminated food or water^9^.

The parasite occurs mainly in the northern hemisphere's temperate and cold climate zones. Generally thought that the cold temperature and high humidity mainly enhance its geographical range and present European expansion^10,11^. This contradicts with observations from the Balkans in recent years. Despite the warming climate and the sub-Mediterranean or Mediterranean climatic conditions, an intensive spread can be seen in the wild canine population in the warmer regions of Europe^5,7,12,13^. This phenomenon also manifests in human alveolar echinococcosis cases^14–16^.

The ongoing expansion of *E. multilocularis* in Europe needs regular and coordinated surveillance to detect the continuance of the continental spread and its changes. In this work, spatial epidemiology should comprise investigations that imply geographical aspects from design to analysis to promote powerful strategies^17^. The principle of spatial epidemiology, also known as spatial autocorrelation, is formulated in Tobler's first law of geography: "Everything is related to everything else, but near things are more related than distant things"^18^. On the one hand, the conventional linear models, which presume the independence of samples, likely give imprecise results if spatial autocorrelation exists in the observations^17^. On the other hand, this approach could detect those explanatory factors that may impact disease in less extended, and similarly characterised areas. For this reason, we analysed our data by multiscale geographically weighted regression to obtain finer results on a regional scale to detect the main environmental drivers of *E. multilocularis* endemic formed in the western Balkan. It is particularly urgent to carry out such investigations in Hungary because the hunting bags of golden jackals and red foxes show an elevated trend. In the last ten years (2013–2022) the hunting bag density of golden jackal and red fox has increased from 0.02 to 0.16 animals/km^2^ and from 0.64 to 0.91 animals/km^2^, respectively (Supplementary Table S1) (National Game Management Database; http://www.ova.info.hu/vgstat.html, accessed 22/10/2023).

## Results

Of the 391 wild canids, we detected 71 infected animals. The distribution of the samples were summarised in Table S1. The table contains the number of samples by UTM quadrates labelled by grid reference and the central coordinates. Both the morphology and the molecular analysis revealed that all investigated worms were *E. multilocularis*. The observed prevalence was 15.2% in red fox, while the golden jackal showed a higher infection rate (21.1%). The prevalence and mean intensity of the two hosts did not differ statistically (Table 1).

**Table 1:** *E. multilocularis* infection in red fox and golden jackal.

	total	red fox	golden jackal	*p*-value (red fox vs golden jackal)
sample size	391	197	194	
infected	71	30	41	
prevalence (%) (CI95%)	18.2 (14.5–22.5)	15.2 (10.8–21.9)	21.1 (15.9–27.5)	0.1494
mean intensity (CI95%)	874 (515–1720)	867 (418–1590)	897 (407–2550)	0.954

### Spatial scan statistics

**Figure 1:**
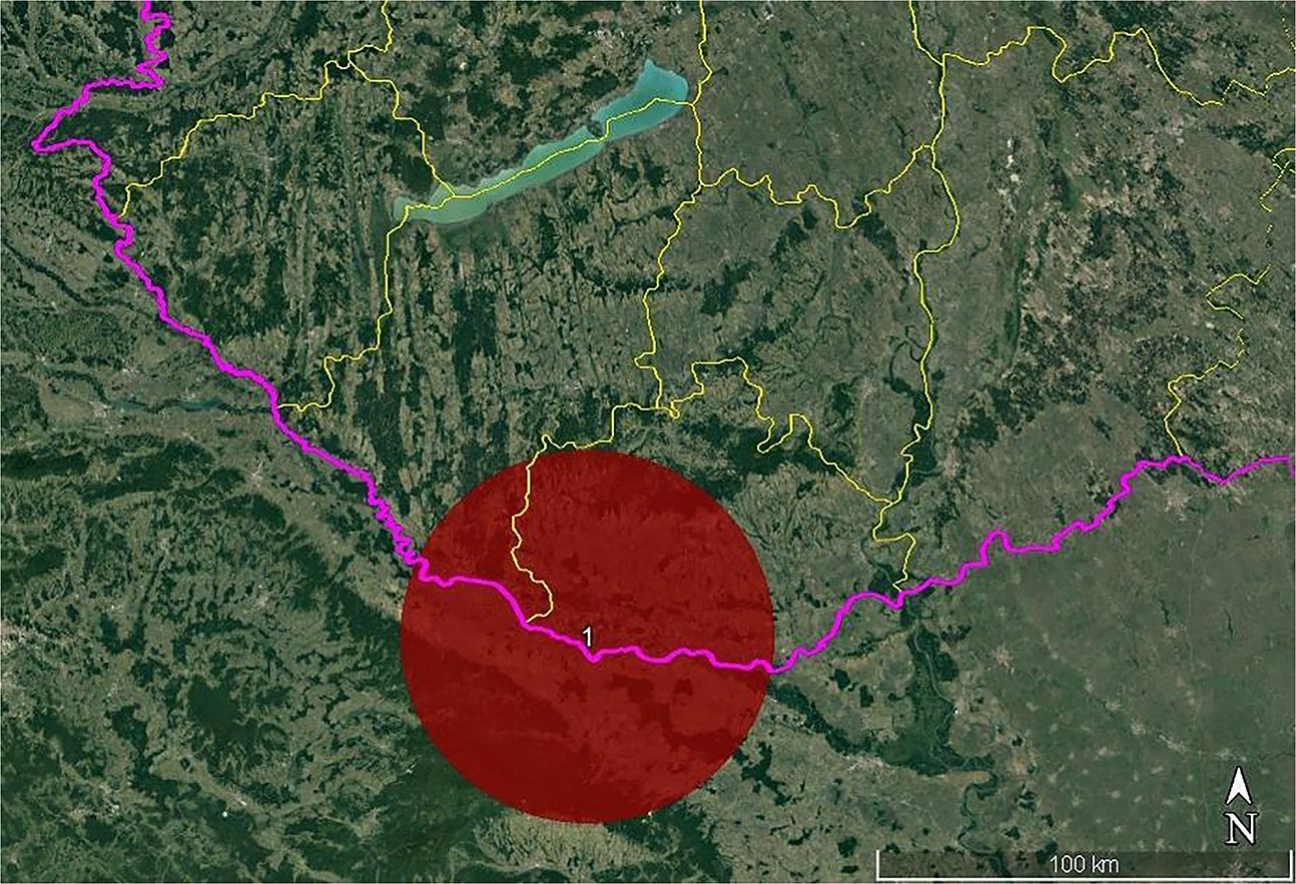
Location of the significant high-rated cluster (Note: red circle = the only significant cluster, purple line = country border, yellow line = county border). (Software used for creation: SaTScan version 10.1 − https://www.satscan.org/, Google Earth Pro version 7.3.6.9345 − https://www.google.com/intl/hu_ALL/earth/about/versions/, and PhotoScape version 3.7. − http://www.photoscape.org/ps/main/download.php?lc=en).

The spatial scan statistics detected one significant and two insignificant clusters. The largest and high-rated cluster (central coordinates: 45.824233 N, 17.843796 E; radius: 44.21 km; relative risk: 3.47; log-likelihood ratio: 16.89; *p* < 0.001) was located in the southern part of the study area and partially covered a part of neighbouring Croatia (Fig. 1).

### Variable selection and ordinary least squares (OLS) model results

**Figure 2:**
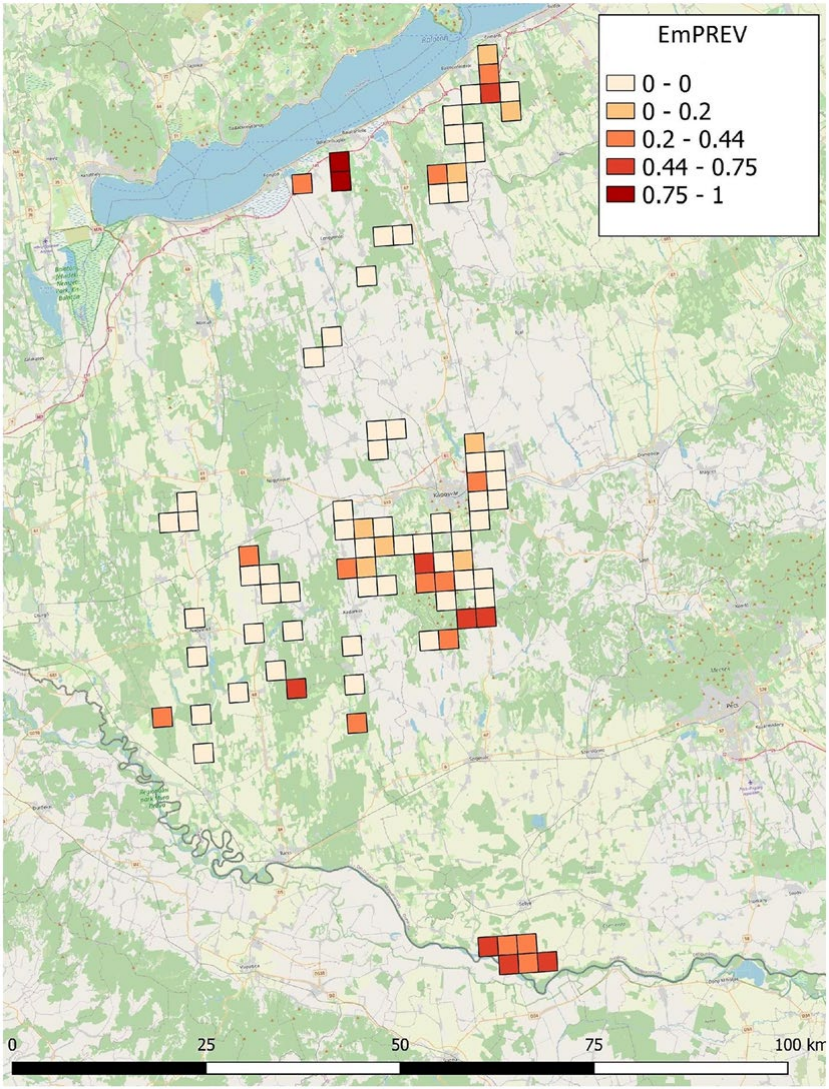
Spatiality of the *E. multilocularis* infection rates. (Software used for creation: QGIS 3.22 Białowieża − https://blog.qgis.org/2021/10/30/qgis-3-22-bialowieza-is-released/).

The spatial autocorrelation analysis confirmed all variables had a significant Moran’s I value (Table 2). The EmPREV also showed a positive spatial autocorrelation (Moran’s I = 0.229, *p* < 0.001). The highly infected sites occurred in the southern (Drava Plain), central (Zselic) and northern parts (Outer Somogy) of the study area (Fig. 2).

**Table 2:** Descriptive statistics and spatial autocorrelation of the dependent and independent variables.

Variable	Mean	SD	Moran’s I	Description
EmPREV	0.145	0.237	0.229***	Prevalence of *E. multilocularis* in UTM quadrates
HUMAN	0.071	0.085	0.219***	Proportion of built environments
GRASS	0.074	0.071	0.230***	Proportion of grasslands (meadows and pastures)
WET	0.027	0.038	0.240**	Proportion of temporary water-covered areas
AGRO	0.359	0.219	0.301***	Proportion of agricultural areas (cropland, orchard, bio-energy plantation)
FOREST	0.465	0.261	0.296***	Proportion of forests
WATER	0.004	0.010	0.111*	Proportion of surface waters (lake, river)
PATCH	7.574	8.521	0.233***	Number of fragments
MAT	11.8	0.191	0.496***	Mean annual temperature (°C)
MAP	704.31	44.011	0.925***	Mean annual precipitation (mm)
DD > 18	477.92	28.25	0.385***	Degree-days above 18 °C
DD < 0	160.34	16.43	0.772***	Degree-days below 0 °C
FFP	216.82	3.07	0.523***	Frost free period (days)
TAVE_cold	1.45	0.331	0.716***	Mean annual temperature of the coldest quarter of the year (°C)
TAVE_warm	21.50	0.255	0.371***	Mean annual temperature of the warmest quarter of the year (°C)
PPT_dry	130.08	7.194	0.906***	Mean annual precipitation of the driest quarter of the year (mm)
PPT_wet	223.92	14.19	0.917***	Mean annual precipitation of the wettest quarter of the year (mm)
T_range	30.89	0.166	0.731***	Annual range of temperature (°C)
PPT_SY	0.186	0.014	0.911***	Seasonality of the annual precipitation

Note: *p < 0.05; **p < 0.01; ***p < 0.001.

Avoiding the multicollinearity, we removed ten possible explanatory variables that had higher VIF values than five. The global (OLS) model explained 7.8% of the variance of *E. multilocularis* infection. By this model, we concluded that only wetlands had spatially consistent significant effects on the dependent variable across the study area (Table 3).

**Table 3:** OLS results of wild canids' *E. multilocularis* infection.

Variable	Coefficient	SD error	t-statistic	*p*-value	VIF
Intercept	− 0.000	0.104	− 0.000	1.000	NA
GRASS	0.021	0.109	0.192	0.848	1.102
WET	0.391	0.142	2.752	0.006	1.878
AGRO	− 0.036	0.121	− 0.296	0.767	1.373
WATER	0.065	0.122	0.532	0.595	1.392
PATCH	0.097	0.119	0.817	0.414	1.325
MAP	0.092	0.187	0.494	0.621	3.255
PPT_SY	− 0.096	0.165	− 0.579	0.563	2.552
MAT	− 0.093	0.153	− 0.609	0.543	2.195

GRASS: grasslands, WET: temporary water-covered areas, AGRO: agricultural areas, WATER: surface waters, PATCH: number of fragments, MAP: mean annual precipitation, PPT_SY: seasonality of the annual precipitation, MAT: mean annual temperature.

During the model analysis, the residual sum of square (RSS) of the OLS showed significant spatial autocorrelation (Moran’s I = 0.092, *p* = 0.042). This output suggested that the correlations between the explained variable and explanatory variables are spatially heterogeneous in the global model. Therefore this model either overestimated or underestimated the prevalence in some areas. For this reason, we developed geographically weighted regression (GWR) and multiscale geographically weighted regression (MGWR) models to explore the local spatial connections.

Both the GWR and MGWR models have substantially improved from the OLS model. These local models explained a more considerable proportion of the variance than the basic global one. GWR and MGWR models had 0.376 and 0.42 adjusted R^2^. In the case of the other performing criteria, the MGWR displayed the best fitting. The two local models produced the random distribution of RSS, and these results indicated both models have effectively alleviated the RSS clustering confirmed in OLS (Table 4). Supplementary Table S2 comprises details on model building. It shows the findings of both the global (OLS) and local (GWR and MGWR) models.

**Table 4:** Comparison of the OLS, GWR and MGWR models by the performing criteria.

Performing criteria	OLS	GWR	MGWR
RSS	72.775	43.569	39.509
RSS Moran’s I (*p*-value)	0.093 (0.042)	− 0.102 (0.081)	− 0.077 (0.155)
AICc	254.257	232.843	229.754
Adj. R^2^	0.078	0.376	0.42

**Figure 3:**
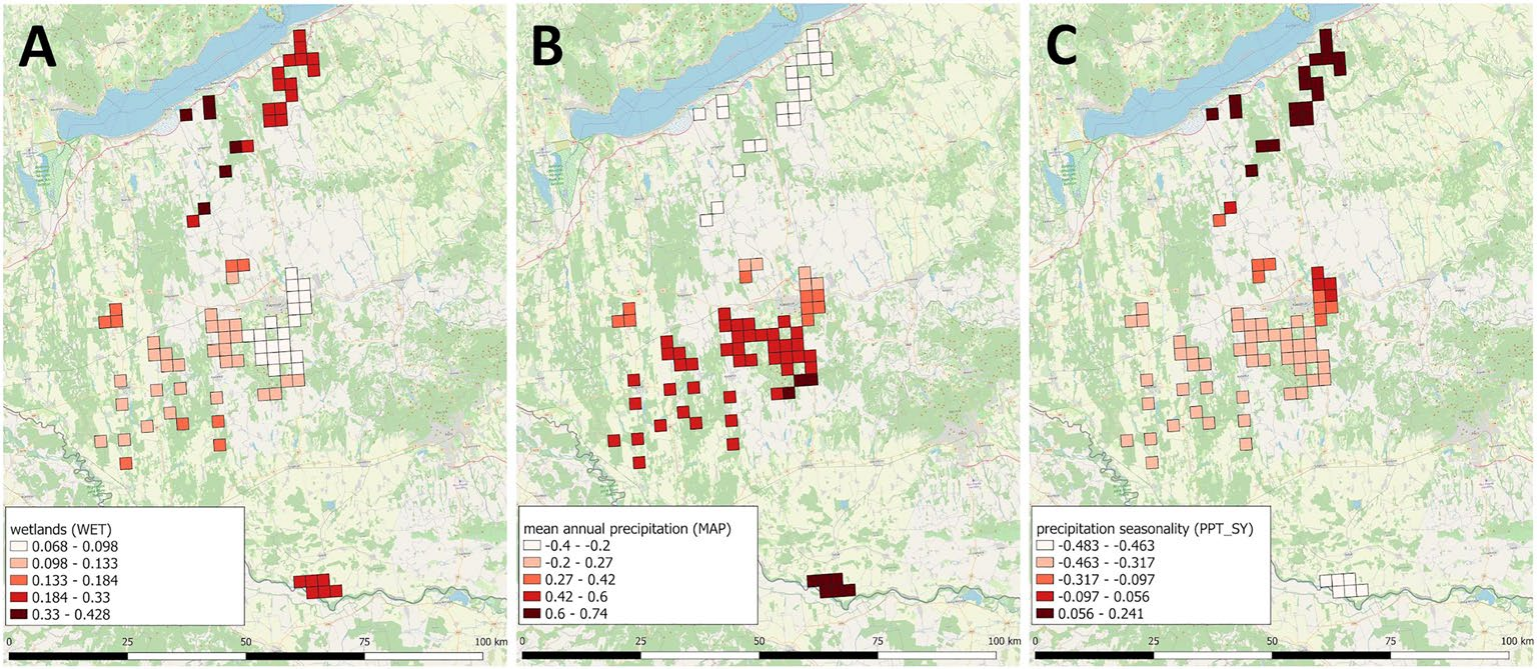
Spatial mapping of the significant drivers. Figure presents the spatial distribution of mean coefficients of WET (**A**), MAP (**B**), PPT_SY (**C**) variables. (Software used for creation: QGIS 3.22 Białowieża − https://blog.qgis.org/2021/10/30/qgis-3-22-bialowieza-is-released/, and PhotoScape version 3.7 − http://www.photoscape.org/ps/main/download.php?lc=en).

**Figure 4:**
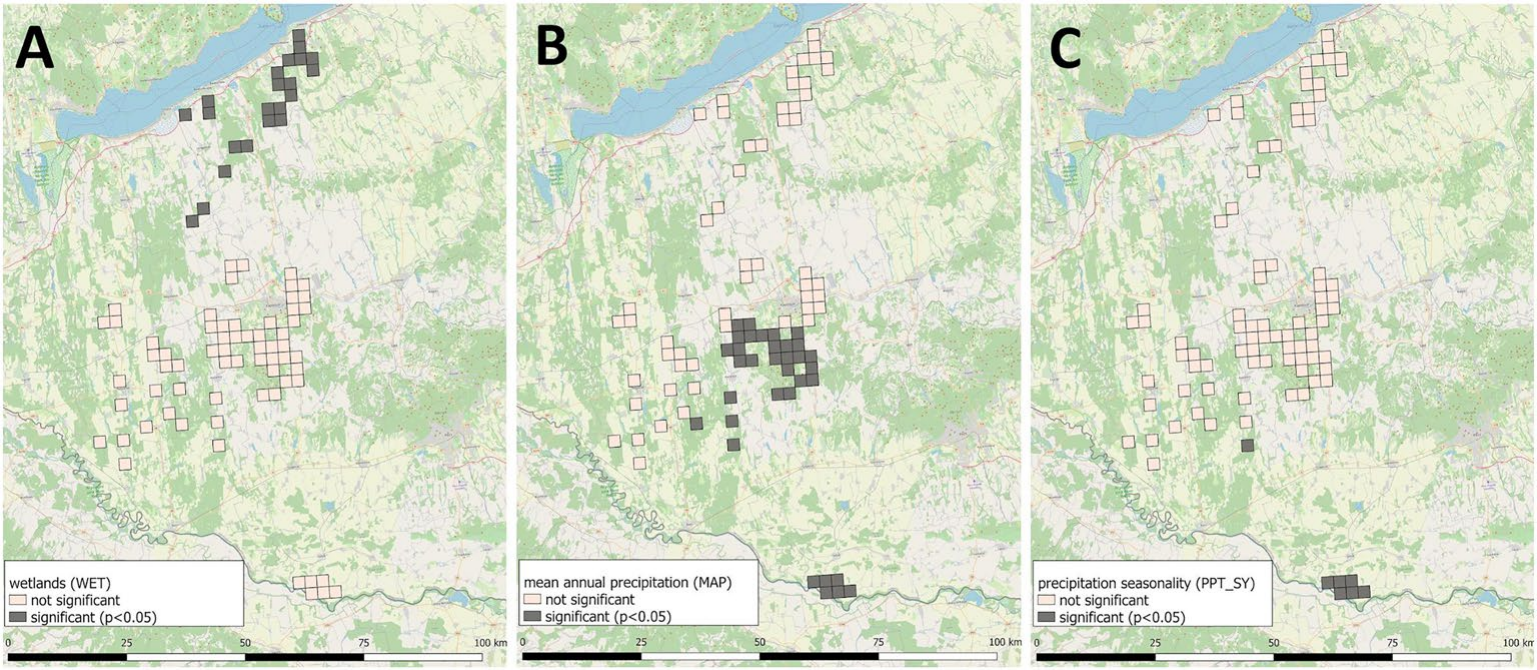
Spatial variation of *p*-values of the significant global (WET: **A**) and local drivers (MAP: **B**, PPT_SY: **C**) obtained from a multiscale geographically weighted regression model. (Software used for creation: QGIS 3.22 Białowieża − https://blog.qgis.org/2021/10/30/qgis-3-22-bialowieza-is-released/, and PhotoScape version 3.7 − http://www.photoscape.org/ps/main/download.php?lc=en).

Based on the results of the three models, we selected the MGWR model for the analysis. This approach performed best in the spatiality of *E. multilocularis* infection in the study area. Three explanatory variables (WET, MAP, PPT_SY) proved significant in the final model. Except for the wetlands, which had a positive global effect, the other two drivers had only non-stationary local effects. The effect of WET, as a global driver, could be seen across the study area. The results confirmed that locally only the MAP and PPT_SY could influence the *E. multilocularis* prevalence. The precipitation had significant positive effects, while its seasonality had a negative relationship with the infection level (Figs. 3, 4, Supplementary Figure S1–S6).

The multiscale approach allows the alterable bandwidth selection during the MGWR model building. Since the bandwidths of the significant explanatory variables were smaller than the amount of the analysed UTM quadrates (n = 87), it was indicated that the influence of certain variables on the prevalence was considerably localised (Table 5). The smaller bandwidths announced a greater variation in a smaller area. For these reasons, we concluded that MAP (bandwidth = 70) and PPT_SY (bandwidth = 74) had only localised effects rather than global.

**Table 5:** MGWR results of wild canids’ *E. multilocularis* infection.

Variable	Mean	SD error	Min	Median	Max	Bandwidth	Monte Carlo *p*-value
Intercept	− 0.273	0.019	− 0.292	− 0.279	− 0.210	85	0.915
GRASS	0.116	0.137	− 0.088	0.158	0.285	67	0.418
WET	0.175	0.098	0.068	0.126	0.428	70	0.711
AGRO	0.090	0.027	0.041	0.094	0.162	81	0.623
WATER	0.072	0.203	− 0.092	− 0.040	0.567	74	0.135
PATCH	0.130	0.146	− 0.149	0.165	0.401	62	0.247
MAP	0.285	0.391	− 0.398	0.491	0.745	70	0.008
PPT_SY	− 0.211	0.271	− 0.483	− 0.381	0.241	74	0.014
MAT	− 0.187	0.062	− 0.288	− 0.190	0.005	72	0.414

## Discussion

*Echinococcus multilocularis* is generally assumed to prefer cool and humid climates ^11^, though its recent range extends to areas with warmer and drier climatic conditions^7,12,13,16^. In this study, we investigated the southwestern part of Hungary where the parasite forms a local endemic^7^. This area can hardly be labelled as cool and humid. Most of the area is under the influence of sub-Mediterranean climate with an arid region in the northeastern part^19^. In this study, we applied geospatial regression models to resolve the contradiction between the recently known environmental demands of the parasite and its current range of occurrence.

Within the investigated area, three microregions seemed to accumulate *E. multilocularis* infection of wild carnivores. The highest prevalence geospatial units turned up within the driest and warmest part of the study area. The continuously very low annual precipitation and very hot summers of Outer Somogy are conspicuous in the dominance of drought tolerant Turkey oak forest stands^20^. Despite the extreme drought periods that characterise this microregion, our findings suggest that an *E. multilocularis* hot spot is forming here. The next most infected area was found by the southern country border, on the Drava Plain. This area is very warm with strong Mediterranean impact, even though the local climate is humid due to the effect of the river and its catchment^19^. Among the detected hot spots, the Zselic area resembles mostly the historical range of *E. multilocularis*. In this microregion, within the globally prevailing sub-Mediterranean climate impact, small sub-mountainous microclimatic patches can be found in deep valleys of the area. As a consequence, azonal European beech stands form a smaller part of the forest coverage^19^. Notwithstanding, the number of high prevalence units were not as high as it was expected regarding the apparently near-ideal climatic conditions of this area.

Analysing the geographical distribution of infected and non-infected animals, we determined one significant high-rated cluster on the Drava Plain. This finding accords with previous studies, which suggest that the river and its catchment area provide appropriate circumstances for the parasite’s maintenance, in spite of the warm, albeit humid, climatic conditions, which considerably differ from the parasite’s environmental requirements^12,16,21^.

To determine the regional drivers of the parasite’s spread and maintenance, we enumerated geographical and climatic factors that are presumed to influence the life cycle of *E. multilocularis*. After multicollinearity analysis, most of the variables had to be removed and only five geographical and three climatic factors were involved in the global (OLS) model (Table 3), of which only wetlands showed a significant effect on the prevalence of *E. multilocularis*. Notwithstanding, this OLS model could explain 7.8% of the variance, which suggested that real drivers of the endemic remained hidden. Mapping the global model’s results (Fig. 4A), it was clear that better relationships between the dependent variable and the explanatory variables existed in the northernmost and the southernmost parts of the study area, while in the central part, the correlation was very weak.

This finding definitely confirmed that global models, even in such a limited area as the recent study site, tend to average geographically various relationships among variables, thus mask fine local patterns^22^. Simple linear regression models, such as OLS, assume that changes across a study area are universal, therefore these models are unable to focus on relationships with local relevance^23^. For this reason, we applied the GWR model, which can analyse the local variations of relationships with a single bandwidth. This model relaxed the OLS model’s assumption that the relationship between the explanatory and the response variables is spatially homogeneous^24^. This model provided a better explanation rate for the variance, whereas it increased from 7.8 to 37.6%. The GWR model assumes that all independent variables influence the dependent variable within the same spatial scale, thus ignoring local multicollinearity and causing overfitting and less reliable parameter estimates^24^.

This shortcoming can be eliminated by the application of the MGWR model, which involves the hypothesis that the influence of an independent variable can vary in a different spatial scale than another one’s. For this reason, the MGWR model computes a covariate-specific, separate optimal bandwidth for each independent variable^23,24^. By applying the MGWR model, we achieved the best estimation for the determinants of *E. multilocularis* infection in wild carnivores within the study area. This model could explain 42.0% of the variance and the AICc showed further decrease (Table 4). The original bandwidth, 87 UTM quadrates, decreased in all investigated independent variables as a result of bandwidth optimization by MGWR model (Table 5).

In this final model, mean annual precipitation (MAP) and the seasonality of the precipitation (PPT_SY) proved significant locally relevant drivers of the endemic, besides the globally affecting wetland (WET) land cover. Mapping the two, locally influential factors showed that mean annual precipitation enhances the epidemic in a humid climate, while the seasonality of the precipitation has an opposite effect in the same areas. Among the hot and dry climatic conditions, these two factors impact reversely, though with very low β-coefficients (Fig. 4B,C). Analysing the strength of the relationships between *E. multilocularis* prevalence and the explanatory variables spatially, it is confirmed that the effect of the wetlands was significant in Outer Somogy exclusively, and not elsewhere. In the case of the mean annual precipitation, the positive effect proved significant in both the cool and the warm humid microregions. Though, the negative effect of precipitation seasonality is confirmed to be significant on the Drava Plain, among the warm and humid climatic conditions.

Contrary to the general assumption that the spread of *E. multilocularis* depends on cold and humid climatic conditions, our study revealed an infection focus within the hottest and driest part of the study area. Geospatial analysis confirmed that those UTM quadrates accumulate infection in this focus, which are characterised by wetlands. By the classification of the European Union’s Mapping and Assessment of Ecosystems and their Services (MAES), wetlands are not surface waters but habitats where the groundwater level can reach the soil surface at least once annually^25^. Wet grasslands and alder carrs are the most prominent representatives of this type of land cover. In some parts of Outer Somogy, the effect of Lake Balaton prevails, whereas the runoff waters and groundwater flow systems of the Somogy Hills discharge around the lake^26^. The high level of groundwater creates wet habitat patches, especially in the valleys between hillsides.

A very similar phenomenon can be observed in the valleys of the Somogy Hills^27,28^ and in the Drava Basin^29^. The stronger appreciation of wetlands in Outer Somogy might be due to the extremely hot and dry climate of this microregion. In these conditions, the balanced microclimate of wetlands provides some protection against the extremities of the surroundings. The higher levels of groundwater transport heat^26^, which can compensate for the harsh effect of hot weather. The direct factor, which benefits from the wetlands, cannot be determined by this research. The plentiful food sources of wetlands as a result of balanced water supply provide appropriate habitats for rodents during the summer. These small mammals, especially field vole *(Microtus arvalis)*, play the role of intermediate hosts of *E. multilocularis*^2^. Undisturbed grasslands provide shelter, nesting site and food source for these animals^30,31^. On the other hand, soil surface humidity, sunlight screening by dense vegetation, and uniform temperature might enhance the egg survival of *E. multilocularis* in hot and dry periods. These hypotheses need further research to clarify.

In the two humid climate areas, geospatial analysis evaluated the effect of wetlands less important in *E. multilocularis* epidemiology. It is probable that the advantageous microclimatic effect of wetlands proves less outstanding in an a priori advantageous environment. In these two focuses, the geospatial analysis of locally acting environmental factors confirmed that mean annual precipitation and the lack of its seasonality, which means consistent humidity all year round, strengthen the endemic. This accords with previous findings, which pointed out the central importance of humidity in the parasite’s maintenance^32,33^.

On the other hand, the cold preference of *E. multilocularis* was not supported by our geospatial investigation. Within the study site, the high-rated cluster was found in the southernmost part with a strong Mediterranean climatic effect. Though the coolest microregion also proved to be an infection focus, its relevance was evaluated subordinate to the Drava Plain focus. The central importance of the Drava Basin is in accordance with Croatian studies, which confirmed positive cases by the river and within its catchment area in carnivores and in human patients^12,16^. These experiences on the spread of *E. multilocularis* infection in warm, albeit humid, climatic conditions query its strong demand for cool environments^11^.

This study did not investigate population density of the definitive and the intermediate hosts, though both have a relevant influence on the epidemiology of echinococcosis. Neither types of hosts have direct data on population size within the study area. In the case of carnivores, hunting bag data can be used as an indicator of trends in population change^34–36^. The data collected in a national database show an explicit increase during the previous decade (Supplementary Table S1). Based on these data, and the findings of others’ studies^37^, the population of golden jackals should be 2500–15,000 individuals in the two counties concerned by our study. The fox population both in Hungary and the study site also shows increase, though with deceleration (Supplementary Table S1). This latter trend is different from Europe-wide population trends in red fox^36^, which needs further research, especially in the view of distinct trends of echinococcosis in the study region.

The abundance of potential intermediate host species is feasible to estimate by indirect methods. In central eastern European agricultural ecosystems, burrow index determination is applied to forecast the expected crop damage caused by the common vole *(Microtus arvalis)*. By this method, the researchers count the number of burrow entrances per ha^31,38,39^. Another option is investigation of rodent specialist raptors’ diet. Owl species^40–43^ and diurnal smaller birds of prey, such as kestrel *(Falco tinnuculus)*^44^ provide detailed data on the composition of rodent fauna of a certain region. The pellets regurgitated by the birds contain the remnants of the rodent species according to their abundance in the environment^40,43,44^.

In the south Transdanubian region of Hungary, where the recent study was carried out, barn owl *(Tyto alba)* is one of the most important vole specialist raptors. In a study between 2015 and 2019, the researchers established that the relative abundance of common vole in the diet of barn owls is 13–94%^41^. Another study found that in semi-natural and agricultural habitats, the frequency of occurrence of common vole in the birds’ diet was 35.6% and 57.7%, respectively. Meanwhile, the occurrence of water vole *(Arvicola amphibius syn. terrestris)* was 1.1–1.2% in the owl pellets. The study highlighted the importance of open lowlands, farmlands, and agricultural intensification in common vole distribution. The loss of landscape heterogeneity resulted in vole dominance both in the habitat and in the pellets^42^. In the Alps, an opposite phenomenon is observed as habitat fragmentation enhances the population expansion of Alpine rodent species resulting in exacerbation of *E. multilocularis* epizootic^34^.

Based on burrow counting and pellet analysis, 2014^41^, 2016^38^, 2017^31^, and 2019^38^ are labelled as common vole outbreak years. Small rodent populations have cyclic increase, peak and crash periods every 2–5 years. However the phenomenon is extensively studied, the exact explanation is still to be found^45,46^. This fluctuation in vole population is synchronous within a large area^38^. The impact of synchronicity can extend for 300 km^43^. During the outbreak events, the population density increases by 2–3 orders of magnitude from the low density phase. The less than one vole per ha under-crash abundance can grow to more than 1000^47^, or even 2000^45^, individuals per ha. This extremely high density of voles can be experienced mostly in fields of perennial crops, such as alfalfa and grasslands^39,45^. These types of land-use are characteristic for those agroecosystems, where the groundwater level is high. Therefore wetlands can support vole populations because of their limited opportunities for agricultural utilisation. Besides land-use, warming climate also increases the quality of vole habitats, which is expected to result in population expansion of these small mammals^47^.

The parasitological method that we applied during this study is not suitable for investigating live animals, therefore it cannot be used for monitoring protected animals, such as grey wolves *(Canis lupus)*. In nature conservation areas and/or in the case of species with nature conservation concern, necropsy of hunter-harvested carcasses is not an option. In these circumstances, faecal samples should be used for estimation of *E. multilocularis* prevalence in wild carnivores^48^.

In this study, we applied GWR and MGWR models to determine locally influential drivers of *E. multilocularis* endemic of the South Transdanubian region of Hungary. These models are able to reveal the subtle local patterns of relationships between explanatory variables and the dependent variable^22,23^. These models are appropriate for analysing epidemiological processes, which have determinants distributed unevenly across space^49^. In spite of their benefit for final scale analysis, these methods have not been introduced into parasitology, yet. With global-scale investigations, *E. multilocularis* is forecasted to spread to cold parts of Europe and expected to become extinct from the southern parts of the continent due to the climate warming^11^. In the lack of fine-scale analysis, western Balkan spread of the parasite is hardly explicable. Application of GWR and MGWR models to a warm climate range of *E. multilocularis* suggested that microclimatic effects might compensate for the disadvantages of an explicitly non-Alpine climate. A long-term follow-up of the western Balkan endemic is needed to evaluate the effects of global warming on *E. multilocularis* survival and life cycle completion. This study calls attention to the importance of fine-scale analysis and locally acting environmental factors, which can delay the expected epidemic fade-out.

Despite Europe's increasingly warm climate, there is clear evidence of the rapid spread of *E. multilocularis*. This is also true in warmer sub-Mediterranean and Mediterranean regions, where average annual temperatures are higher, and rainfall is lower than in the central and northern parts of the continent. For this reason, it is necessary to apply the spatial modelling of disease transmission at the fine-scale analysis to identify possible drivers that may influence infection rates in host species. This research exploited the advantages of a spatial modelling framework to unfold the relationship between infection prevalence and a set of land use and climatic variables in the Hungarian part of the western Balkan region. The applied global (OLS) and local (MGWR) regression models were utilised and compared to elucidate the spatial variance observed in the infection rate. Overall, the global model did not adequately represent the observed spatial variation (adj. R^2^ = 0.078), and the goodness-of-fit of this model was also much larger (AICc = 254.257). The MGWR provided the best overall fit (adj. R^2^ = 0.42, AICc = 229.754) and was able to capture local patterns. For this reason, we concluded the MGWR was a useful tool for modelling the spatial variation at local geographic scales. To our knowledge, this is the first study to model *E. multilocularis* infection level in wild canid hosts at a smaller regional scale. Although the results of this analysis could allow a closer focus on prevalence and land cover (extension of wetlands) and environmental covariates (mean annual precipitation and precipitation seasonality), further investigations are needed to evaluate the effects of global warming on *E. multilocularis* survival and life cycle completion in the western Balkan endemic.

## Material and methods

### Sampling sites

**Figure 5:**
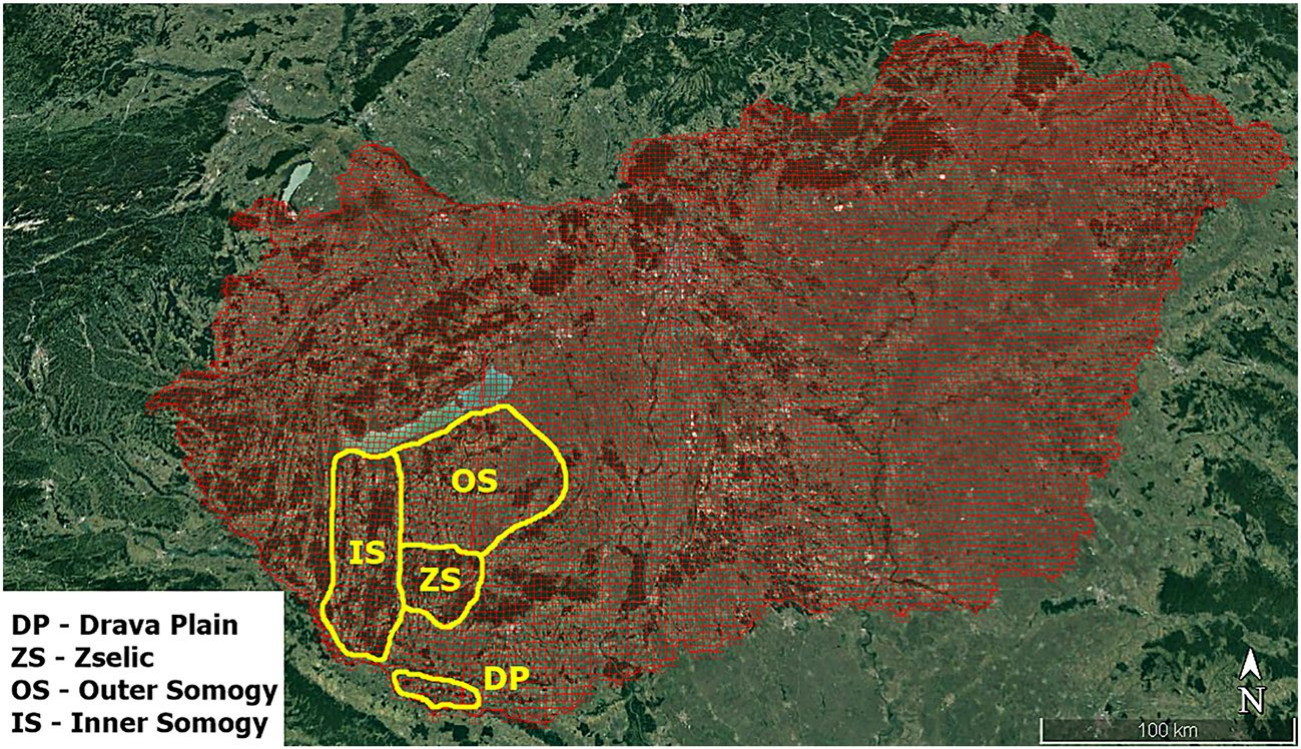
Localisation of study areas. Localisation of study areas. The country map is covered by the 2.5 × 2.5 km Universal Transverse Mercator (UTM) system grids. (Software used for creation: Google Earth Pro version 7.3.6.9345 − https://www.google.com/intl/hu_ALL/earth/about/versions/, and PhotoScape version 3.7. − http://www.photoscape.org/ps/main/download.php?lc=en).

From January 2021 to December 2022, legally hunted red fox (n = 197) and golden jackal (n = 194) specimens were collected from the Transdanubian region of Hungary (Baranya County and Somogy County). The samples originated from four different, well-separated sites: Drava Plain (DP), Zselic (ZS), Outer Somogy (OS) and Inner Somogy (IS) (Fig. 5).

In a previous study, Lanszki et al. found that the jackal density varied between 0.05 and 0.28 groups/km^2^ in our study area^50^, while the average group size assessed 4–5 animals in a Hungarian study, conducted in the Transdanubian region^51^. Based on these bioacoustic and scat analysed data, the assumed density could have been about 0.2–1.12 individuals per km^2^. The golden jackal hunting bag statistics confirmed that in Baranya and Somogy County, the number of harvested golden jackal greatly exceeded national average (Supplementary Table S1) (National Game Management Database (http://www.ova.info.hu/vgstat.html, accessed 22/10/2023).

In the area, three climates combine and influence the general climatic pattern. The continental climate zone is in the northeastern part (OS), the Mediterranean in the southern part (DP), and the influence of the Atlantic climate zone can be detected in the west (IS)^20^. According to the European geobotanical classification, the sampling sites are rated into the submontane oak hornbeam woodlands and thermophilous oak woodlands with open steppe oak woodlands and riparian vegetation^20,52^.

### Drava Plain

This site is located on the floodplain of the lower section of the Drava River. The area is characterised by a considerable coverage of constant water flows (rivers, creeks, ditches, oxbows). The main utilisation type of land is agriculture; its proportion is approximately 75% (including arable lands, meadows and wood pastures). The average elevation of the sandy soiled surface is 96–110 m above sea level (a.s.l.). The long-term mean annual temperature is 10.8 °C, while the mean annual precipitation is approximately 750 mm^28^.

### Zselic

This hilly area is the most forested landscape in South Transdanubia. The two dominant land covering categories are the forests and agricultural areas, about 60% and 37%, respectively. The agricultural fields (viz. grasslands, pastures, arable lands and old orchards) provide a mosaic landscape structure, mainly on the site's periphery. The most frequent forest-covered habitats are swamp woodlands, pedunculate oak-hornbeam woodlands and sessile oak-hornbeam woodlands. In the steep valleys, submontane mesophilic beech forests can be found.

The proportion of watercourses is relatively high, but most are intermittent. Thus their base runoff mainly depends on annual precipitation. On the site, brown forest soils with clay illuviation are typical. The average elevation of the hills is 220–250 m a.s.l. Its climate is diversified, generally sub-Mediterranean, with some submontane habitat patches. The mean annual temperature ranges 10.2–10.7 °C. The mean annual precipitation varied between 630 and 720 mm^19^.

### Inner Somogy

Inner Somogy has diverse landscape characteristics. The proportion of forests and agricultural areas (arable lands, grasslands, wood pastures) is harmonised, about 40% and 50%, respectively. There are several woodland types in the area, its western part is characterised by riverine swamps, and in the eastern part, mainly oak-elm-ash forests exist. A high extension of fishponds and constant water flows (creeks and ditches) dominate the remaining part. The mean elevation is 173 m a.s.l. The climate is similar to Drava Plain (mean annual temperature: 10.7 °C, mean annual precipitation: 715–725 mm)^19^.

### Outer Somogy

This sampling site is bordered by Lake Balaton (North), Kapos River (South), Sió Channel (East) and Inner Somogy (west). This area has the smallest woodland proportion (approximately 30%) among the investigated regions. The dominant land covering categories are arable lands, pastures, orchards, and the human settlements found on the shore of Lake Balaton. In the valleys, usually constant watercourses, swamps and marshes can be found. The site’s surface shows the highest difference in elevation 94–316 m a.s.l. (mean 183 m). Outer Somogy is the driest site; its mean annual precipitation is 640 mm with a 10.8 °C mean annual temperature^19^.

### Parasitological and molecular analysis

All the animals were culled during hunting events or for predator depopulation plans permitted by the local hunting authorities. We transferred the hunter-harvested carcasses to the Hungarian University of Agriculture and Life Sciences laboratory, then froze them at − 80 °C for five days—for biosecurity. During the necropsy, we opened the small intestine and washed its content and its mucosal layer content into a plastic jar. After a thorough homogenisation, the suspension was sedimented for at least 10 min, the supernatant was decanted, and the residual was homogenised again with lukewarm tap water. This procedure was repeated until the supernatant became clear for worm detection. The parasites were collected using a stereo-microscope at magnification 40× . All worms were counted if their number did not exceed 100. Above 100 worms, we filled the jar again up to 1 L, and after a thorough stirring, we took two 100 mL subsamples. In this case, the total worm burden was calculated from the subsample count by multiplying the worm numbers by 5. The overall and within species prevalence and mean intensity were calculated. All calculations were conducted using the online version of Quantitative Parasitology (Qpweb) software^53^. The collected parasite specimens were placed into 70% alcohol until morphological and molecular identification.

We identified *E. multilocularis* specimens based on the cardinal morphometric characteristics, such as the position of the genital pores of proglottid, ratio of worm length to the terminal proglottids, sac-like uterus^54^. For the final confirmation a multiplex PCR was used with the following primer sequences for taxonomy identification: Cest1: 5′-TGCTGATTTGTTAAAGTTAGTGATC-3′ f and Cest2: 5′-CATAAATCAATGGAAACAACAACAAG-3′ for *E. multilocularis* and Cest4: 5′-GTTTTTGTGTGTTACATTAATAAGGGTG-3’ and Cest5: 5′-GCGGTGTGTACMTGAGCTAAAC-3’ for *E. granulosus* sensu lato. The amplicons were resolved on 2% agarose gel. The 395 bp fragment lengths verified the presence of *E. multilocularis*. The specificity of the applied test was 100%^55^.

### Variable selection

Before selecting the variables, the investigated specimens were ordered into spatial units based on their shooting coordinates. Spatial units were created for statistical analysis^56^. We used 2.5 × 2.5 km Universal Transverse Mercator (UTM) system grids as spatial units. On the one hand, these quadrates’ size (6.25 km^2^) is almost equal to the average home range used by foxes and jackals^57,58^. Based on the assumed ackal group density (0.05–0.28 groups/km^2^) in the study site (see *Sampling site* section), we presumed that at least one group could have lived in an investigated UTM quadrate and used it in whole or partly as a home range. This conjecture was strengthened in a telemetry study, using autocorrelated kernel density estimation (AKDE), wherein was found that golden jackals’ average home range size proved 2.39 km^2^ (50% AKDE) and 11.17 km^2^ (90% AKDE) in southern Hungary and northern Serbia^59^. For foxes, diverse home range sizes were determined in different regions of Europe^34–36^. For comparability of data from different host species, we used a uniform grid for both hosts.

Nevertheless, these UTM quadrates could serve as valid spatial and decisional units, and provided a finer and more effective methodology for spatial analysis^60,61^. For this reason, we inserted all shooting coordinates into grids (n = 87), and after this, we used them as spatial units (Supplementary Table S3). We calculated the *E. multilocularis* prevalence of the UTM grids, and during the geospatial analysis, these values were applied as the dependent variable (EmPREV).

After the mentioned arrangement, we determined the different land cover categories of the UTM quadrate using a 20 × 20 m resolution land cover map (Ecosystem Map of Hungary,

(http://alapterkep.termeszetem.hu/; accessed on 14 February 2023). We chose six different land cover categories and used their calculated proportions in the spatial units as explanatory variables: HUMAN (including buildings, roads, railways, and artificial surfaces); GRASS (including different types of meadows and pastures); WET (including wetlands covered with herbaceous or woody plants); AGRO (including arable lands, orchards, energy plantations); FOREST (including deciduous and coniferous forests in different habitats and composition); and WATER (lakes and watercourses). In every spatial unit, fragmentation was also evaluated. In this case, the main roads, highways, settlements, lakes and rivers were chosen as barriers that can produce more or less isolated segments of habitats (patches) and impede the movement of the animals and, therefore, may influence the spread of a certain pathogen. We use the number of patches (PATCH) as an independent variable. For the data extraction, the QGIS (version 3.22—Białowieża) and the FragScape plug-in were applied^62^.

Annual and seasonal climatic variables were collected from the ClimatEU software^63^. To obtain the data, we used the central coordinates of the UTM grids. All values were averages gained over a 30 year period (1991–2020). We involved mean annual temperature (MAT), mean annual precipitation (MAP), degree-days above 18 °C (DD > 18), degree-days below 0 °C (DD < 0), and frost-free period (FFP), coldest quarter mean temperature (TAVE_cold), warmest quarter mean temperature (TAVE_warm), driest quarter precipitation (PPT_dry), wettest quarter precipitation (PPT_wet). We calculated two more climatic factors. Temperature annual range (T_range) was formed as the difference between a year's maximum and minimum temperatures. For the calculation of precipitation seasonality (PPT_SY), we used Walsh and Lawler's index:

$$ {\text{SI}}_{{\text{i}}} = \frac{1}{{{\text{R}}_{{\text{i}}} }}\sum\limits_{{{\text{n}} = 1}}^{{{\text{n}} = 1}} {\left| {{\text{x}}_{{{\text{in}}}} - \frac{{{\text{R}}_{{\text{i}}} }}{12}} \right|} $$

where R_i_ *i*s the total annual precipitation of a certain year, and X_in_ is the actual monthly precipitation of month n. The SI_i_ could have seven categories. If its value < 0.19, the precipitation is very equal every month. In the case of extreme seasonality, the index is above 1.2 and all precipitation falls in 1–2 months^64^.

### Scan statistics

Based on the localisation of infected and non-infected animals, we applied scan statistics to expose high- and low-rate clusters for *E. multilocularis* infection. For this reason, we applied SaTScanTM software^65^. We used a purely spatial Bernoulli model. The identified clusters' significance is based on a likelihood ratio test and calculated for 999 Monte Carlo simulations with the maximum cluster size of 50% of the total population for parasites (as cases).

### Global regression model

Before the model buildings, we checked the multicollinearity between the variables and selected all of the variance inflation factor (VIF) that was higher than five^66^. An ordinary least squares (OLS) model was conducted for the global, non-spatial analysis. This model detects and explains the connection between the dependent (EmPREV) and independent variables (chosen land cover and climatic factors)^67^. This model's basic principles are: (1) independence of the observations, and (2) the relation between the explained and explanatory variables does not vary over the study area. In other words, this stationarity means that each predictor variable has the same impact (indicated as β coefficient) in each geographical unit. Although OLS is a generally used practical method, its prediction deteriorates if the relationships between the variables are spatially heterogeneous. After OLS building, we calculated Moran's I to assess the presence of spatial autocorrelation in the residuals. If the parameter is significant, it indicates the presence of spatial autocorrelation, therefore raising the needfulness of spatial regression models such as GWR or MGWR^68^. This analytical step was implemented using GeoDa software^69^.

### Local regression model

We used two approaches for local modelling, the geographically weighted regression (GWR) and the multiscale geographically weighted regression (MGWR). These models are local forms of linear regression models. They can consider site-specific variations and analyse spatially varying relationships^70^.

GWR performs a regression model on each spatial unit and weights different regression parameters across the study area, given a response and a set of predictor variables. During the model building, GWR searches for an optimal bandwidth (number of neighbours) across all covariates and processes at the same spatial scale. Thus in GWR, the regression was estimated for every spatial unit of analysis and provides the predictive local effects. This method can calculate different coefficients for each spatial unit; after mapping the results, powerless and robust (significant and insignificant) relations could be detected^71^.

The general bandwidth in GWR occasionally has its limitations. This disadvantage was dissolved by Yang^72^ and Fotheringham et al.^73^, who suggested and elaborated a multi-bandwidth approach to creating a more accurate and useful method, MGWR. The introduction of varying bandwidths across parameters allowed the model to have an optimal number of neighbours in every spatial unit, consequencing a proper estimation, which can result in a better prediction for all response variables^73^.

The two local models were performed by MGWR 2.2 software^74^. For the best performance, we chose the adaptive square spatial kernel and golden search bandwidth selection to detect the optimal number of neighbours in each variable. The Monte Carlo test was used with 999 repetitions to decide whether the spatial variability of the local estimates is attributable to sampling variation or just a result of other inherent processes.

For model evaluation, we used multi-criteria: adjusted R2 (Adj. R2), corrected Akaike information criterion (AICc), residual sum of square (RSS), and Moran's I of RSS. The larger Adj. R2 indicated that certain models explained a larger variance. The smaller AICc meant the model was more parsimonious, while the smaller RSS indicated a larger variance (similar to Adj. R2), and the model was more fit.

### Supplementary Information

Supplementary Information 1.

Supplementary Information 2.

Supplementary Information 3.

Supplementary Information 4.

Supplementary Information 5.

Supplementary Information 6.

Supplementary Table 1.

Supplementary Table 2.

Supplementary Table 3.

Supplementary Legends.

## Data Availability

The data presented in this study are available on request from the corresponding author.
