# Supplementary figures and images for: Global and local drivers of Echinococcus multilocularis infection in the western Balkan region
Source: Sci Rep. 2023 Dec 1;13:21176. doi: 10.1038/s41598-023-46632-9 (PMC10692075; doi:10.1038/s41598-023-46632-9)

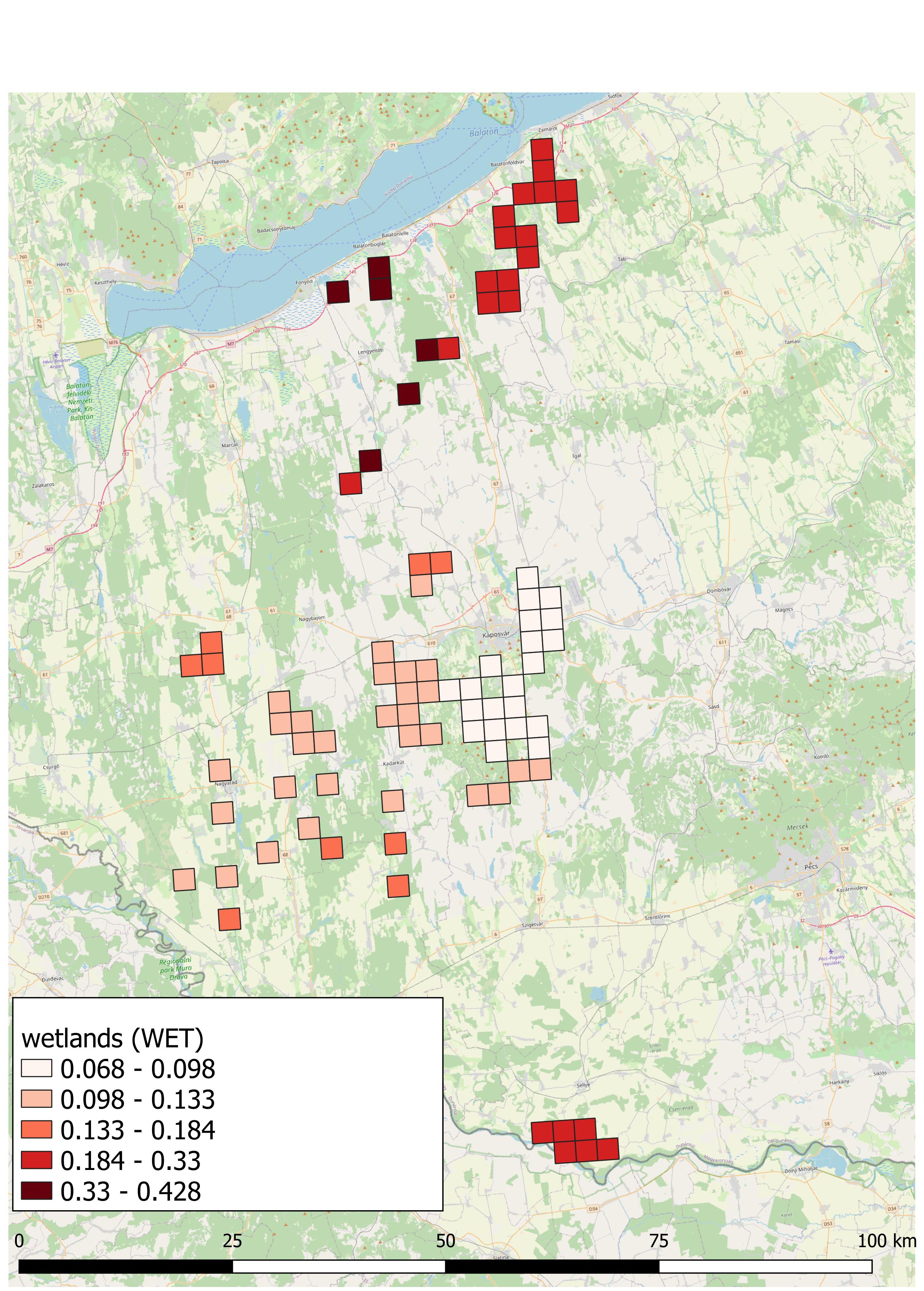

Supplement: Supplementary file 1 — Supplementary Information 1. [file 41598_2023_46632_MOESM1_ESM.jpg]

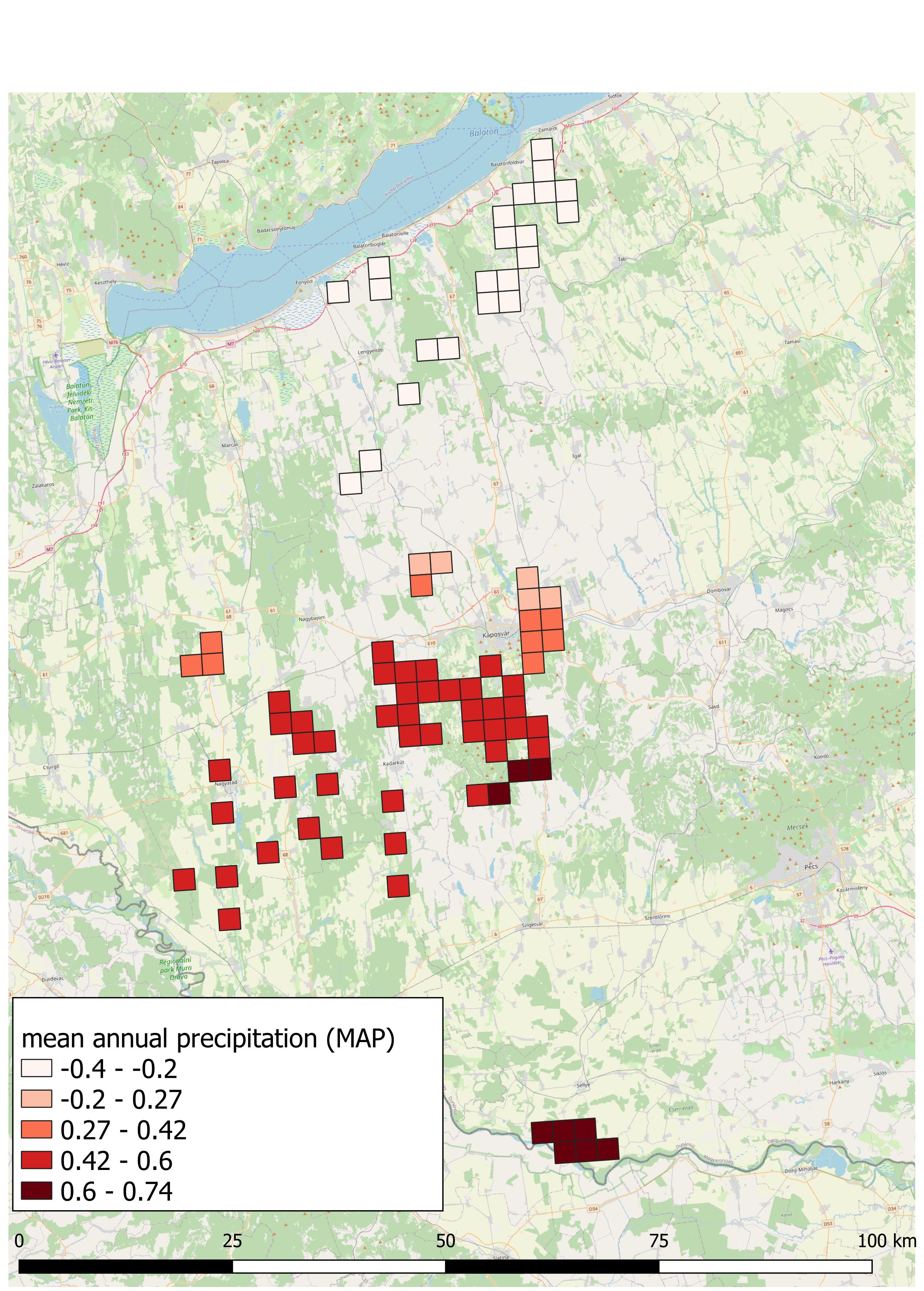

Supplement: Supplementary file 2 — Supplementary Information 2. [file 41598_2023_46632_MOESM2_ESM.jpg]

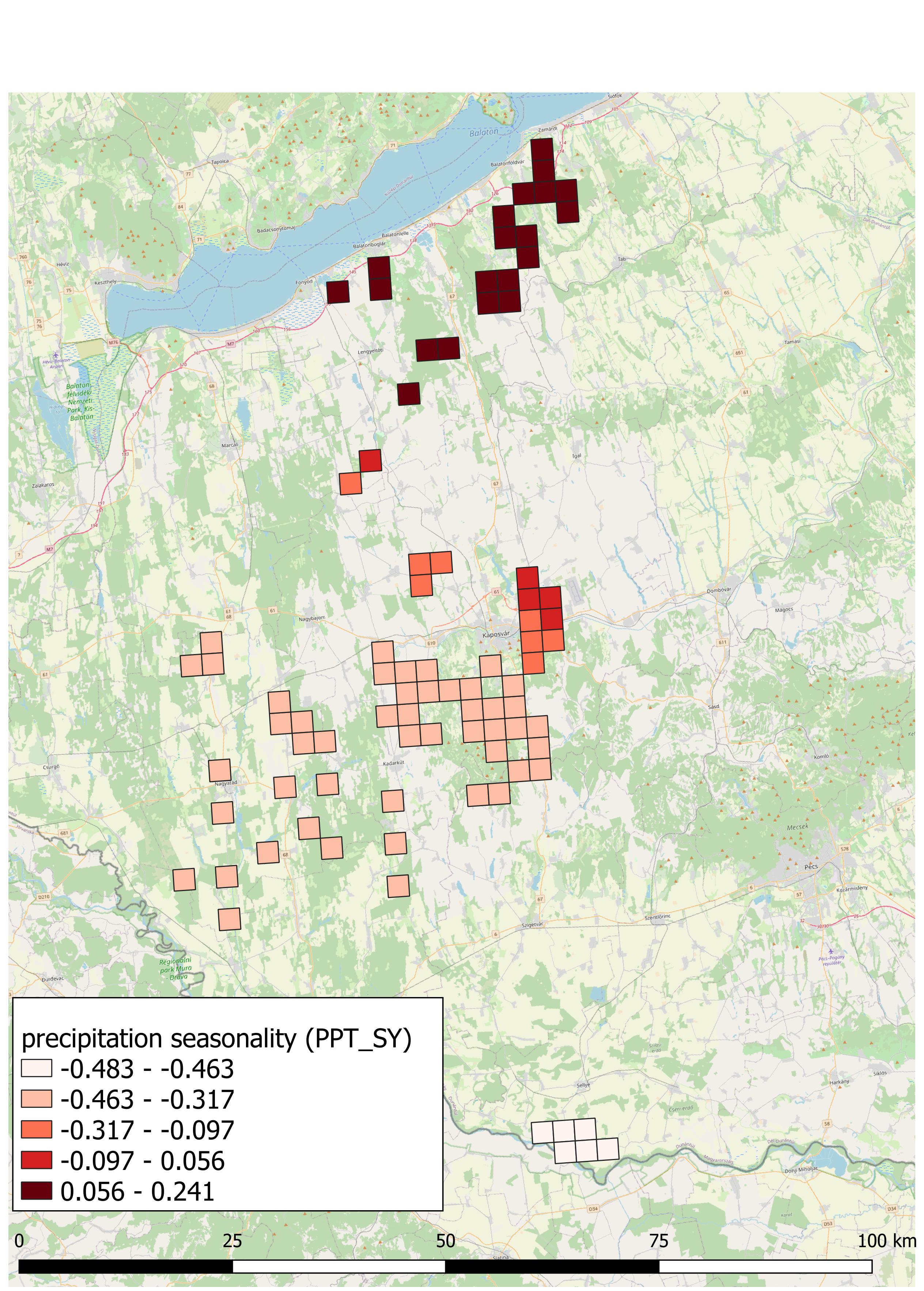

Supplement: Supplementary file 3 — Supplementary Information 3. [file 41598_2023_46632_MOESM3_ESM.jpg]

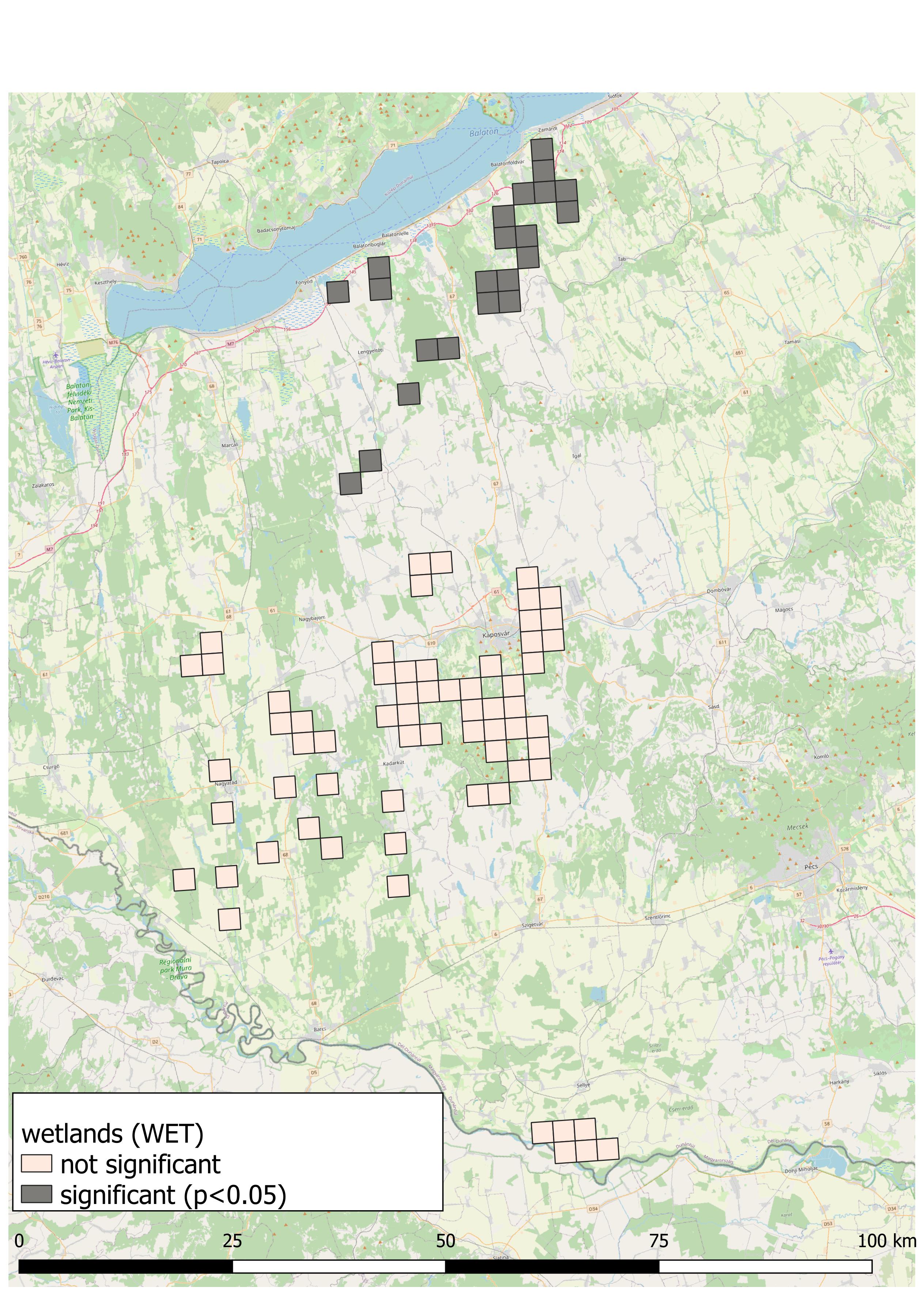

Supplement: Supplementary file 4 — Supplementary Information 4. [file 41598_2023_46632_MOESM4_ESM.jpg]

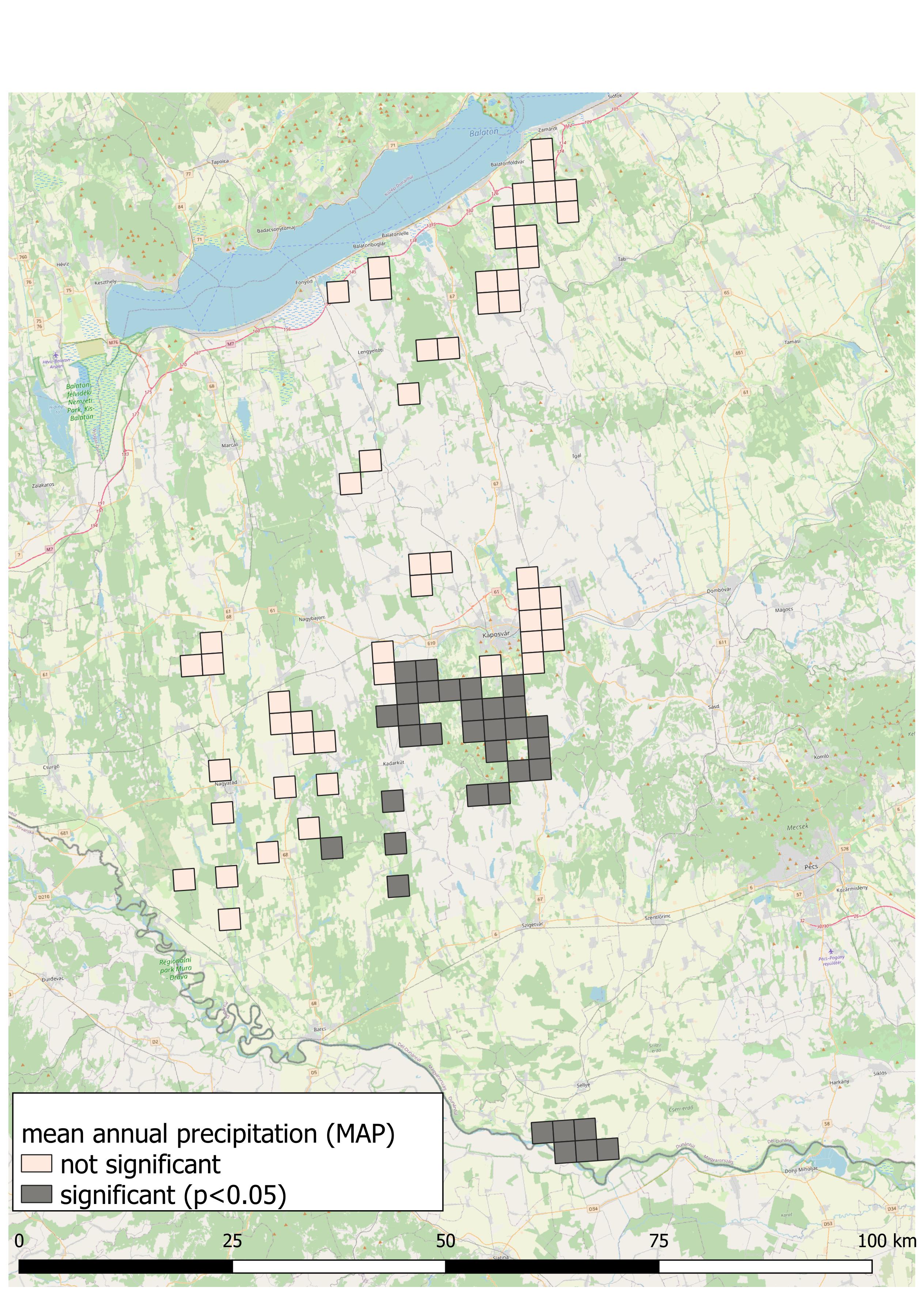

Supplement: Supplementary file 5 — Supplementary Information 5. [file 41598_2023_46632_MOESM5_ESM.jpg]

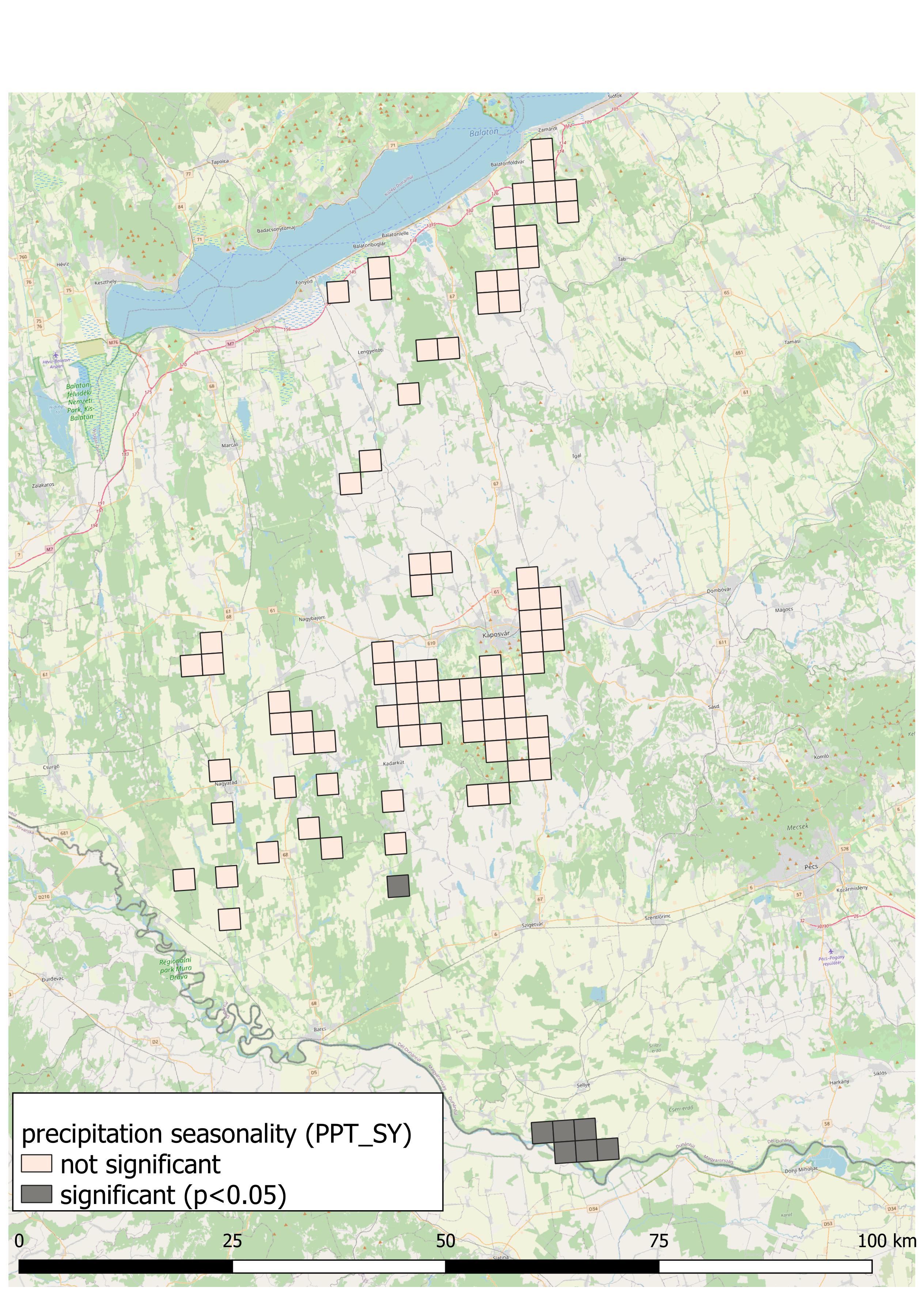

Supplement: Supplementary file 6 — Supplementary Information 6. [file 41598_2023_46632_MOESM6_ESM.jpg]
