# Supplementary material for: Global and local drivers of Echinococcus multilocularis infection in the western Balkan region
Source: Sci Rep. 2023 Dec 1;13:21176. doi: 10.1038/s41598-023-46632-9 (PMC10692075; doi:10.1038/s41598-023-46632-9)
Supplement: Supplementary file 7 — Supplementary Table 1. [file 41598_2023_46632_MOESM7_ESM.pdf]

Global and local drivers of *Echinococcus multilocularis* infection in the western Balkan region

Sibusiso Moloi, Tamás Tari, Tibor Halász, Bence Gallai, Gábor Nagy, Ágnes Csivincsik

Table S1. Hunting bag statistics of the studied areas (Baranya County and Somogy County) and Hungary.

	Hungary		Baranya County		Somogy County	
total area	93030 km²		4430 km²		6065 km²	
hunting year	jackal* (animals/km²)	fox (animals/km²)	jackal (animals/km²)	fox (animals/km²)	jackal (animals/km²)	fox (animals/km²)
2013	1813 (0.02)	59745 (0.64)	452 (0.1)	2477 (0.56)	650 (0.11)	2516 (0.41)
2014	2535 (0.03)	73333 (0.79)	511 (0.12)	2620 (0.59)	946 (0.16)	2656 (0.44)
2015	3267 (0.04)	70125 (0.75)	707 (0.16)	2947 (0.67)	999 (0.16)	2557 (0.42)
2016	4225 (0.05)	69111 (0.74)	785 (0.18)	2722 (0.61)	1441 (0.24)	2876 (0.47)
2017	5831 (0.06)	72694 (0.78)	1043 (0.24)	3168 (0.72)	1668 (0.28)	2965 (0.49)
2018	7873 (0.08)	82687 (0.89)	1502 (0.34)	3041 (0.69)	2069 (0.34)	3984 (0.66)
2019	11283 (0.12)	91285 (0.98)	2014 (0.45)	2646 (0.6)	3378 (0.56)	5766 (0.95)
2020	12126 (0.13)	89439 (0.96)	2036 (0.46)	2882 (0.65)	2936 (0.48)	4328 (0.71)
2021	12620 (0.14)	88696 (0.95)	2033 (0.46)	2625 (0.59)	2710 (0.45)	4201 (0.69)
2022	14831 (0.16)	84537 (0.91)	2092 (0.47)	2736 (0.62)	3225 (0.53)	4253 (0,7)

*annual hunting bag of the particular year, the parenthic number indicates the hunting bag density (harvested animals/km²)
