# Supplementary material for: Global and local drivers of Echinococcus multilocularis infection in the western Balkan region
Source: Sci Rep. 2023 Dec 1;13:21176. doi: 10.1038/s41598-023-46632-9 (PMC10692075; doi:10.1038/s41598-023-46632-9)
Supplement: Supplementary file 8 — Supplementary Table 2. [file 41598_2023_46632_MOESM8_ESM.pdf]

Global and local drivers of Echinococcus multilocularis infection in the western Balkan region

Sibusiso Moloi, Tamás Tari, Tibor Halász, Bence Gallai, Gábor Nagy, Ágnes Csivincsik

Table S2. Detailed results of the global (OLS) and local (GWR and MGWR) models.

MGWR Version: 2.2.1
Released on: 03/20/2020

Model type:	Gaussian
Number of observations:	87
Number of covariates:	9
Dependent variable:	EmPREV
Variable standardization:	On

Global Regression Results

Residual sum of squares:	72.775
Log-likelihood:	-115.681
AIC:	249.362
AICc:	254.257
R2:	0.164
Adj. R2:	0.078

Variable	Est.	SE	t(Est/SE)	p-value
Intercept	-0.000	0.104	-0.000	1.000
GRASS	0.021	0.109	0.192	0.848
WET	0.391	0.142	2.752	0.006
AGRO	-0.036	0.121	-0.296	0.767
WATER	0.065	0.122	0.532	0.595
PATCH	0.097	0.119	0.817	0.414
MAP	0.092	0.187	0.494	0.621
PPT_SY	-0.096	0.165	-0.579	0.563
MAT	-0.093	0.153	-0.609	0.543

Geographically Weighted Regression (GWR) Results

Coordinates type:	Projected
Spatial kernel:	Adaptive bisquare
Criterion for optimal bandwidth:	AICc
Bandwidth used:	78.000
Bandwidth confidence interval (95%):	(69.0, 80.0)

Diagnostic Information

Residual sum of squares:	43.569
Effective number of parameters (trace(S)):	17.017

Degree of freedom (n - trace(S)):	69.983
Sigma estimate:	0.789
Log-likelihood:	-93.365
Degree of Dependency (DoD):	0.857
AIC:	222.764
AICc:	232.843
BIC:	267.191
R2:	0.499
Adj. R2:	0.376
Adj. alpha (95%):	0.026
Adj. critical t value (95%):	2.259

Monte Carlo Test for Spatial Variability

Variable	p-value
Intercept	0.243
GRASS	0.205
WET	0.586
AGRO	0.287
WATER	0.007
PATCH	0.323
MAP	0.003
PPT_SY	0.000
MAT	0.290

Summary Statistics For GWR Parameter Estimates

Variable	Mean	STD	Min	Median	Max
Intercept	-0.179	0.067	-0.417	-0.183	-0.063
GRASS	0.126	0.088	-0.027	0.139	0.261
WET	0.173	0.082	0.057	0.150	0.306
AGRO	0.043	0.063	-0.066	0.052	0.141
WATER	0.119	0.225	-0.095	-0.009	0.556
PATCH	0.135	0.081	-0.007	0.124	0.282
MAP	0.231	0.546	-0.690	0.334	1.065
PPT_SY	-0.218	0.499	-0.981	-0.346	0.551
MAT	-0.182	0.113	-0.391	-0.143	-0.048

Multiscale Geographically Weighted Regression (MGWR) Results

Coordinates type:	Projected
Spatial kernel:	Adaptive bisquare
Criterion for optimal bandwidth:	AICc
Score of change (SOC) type:	Smoothing f
Termination criterion for MGWR:	1.0e-05
Number of iterations used:	34

MGWR bandwidths

Variable	Bandwidth	ENP_j	Adj t-val(95%)	DoD_j
Intercept	85.000	1.077	2.020	0.983
GRASS	67.000	2.614	2.388	0.785
WET	70.000	2.244	2.328	0.819

AGRO	81.000	1.592	2.188	0.896
WATER	74.000	2.424	2.358	0.802
PATCH	62.000	2.206	2.321	0.823
MAP	70.000	2.204	2.320	0.823
PPT_SY	74.000	2.042	2.290	0.840
MAT	72.000	2.249	2.328	0.819

Bandwidth Confidence Intervals (95%)

Intercept	(59.0, 85.0)
GRASS	(59.0, 76.0)
WET	(59.0, 76.0)
AGRO	(59.0, 83.0)
WATER	(59.0, 81.0)
PATCH	(53.0, 70.0)
MAP	(59.0, 76.0)
PPT_SY	(59.0, 81.0)
MAT	(59.0, 76.0)

Monte Carlo Test for Spatial Variability

Variable	p-value
Intercept	0.915
GRASS	0.418
WET	0.711
AGRO	0.623
WATER	0.135
PATCH	0.247
MAP	0.008
PPT_SY	0.014
MAT	0.414

Diagnostic Information

Residual sum of squares:	39.509
Effective number of parameters (trace(S)):	18.651
Degree of freedom (n - trace(S)):	68.349
Sigma estimate:	0.760
Log-likelihood:	-89.110
Degree of Dependency (DoD):	0.837
AIC:	217.521
AICc:	229.754
BIC:	265.979
R2:	0.546
Adj. R2:	0.420

Summary Statistics For MGWR Parameter Estimates

Variable	Mean	STD	Min	Median	Max
Intercept	-0.273	0.019	-0.292	-0.279	-0.210
GRASS	0.116	0.137	-0.088	0.158	0.285
WET	0.175	0.098	0.068	0.126	0.428
AGRO	0.090	0.027	0.041	0.094	0.162
WATER	0.072	0.203	-0.092	-0.040	0.567

PATCH	0.130	0.146	-0.149	0.165	0.401
MAP	0.285	0.391	-0.398	0.491	0.745
PPT_SY	-0.211	0.271	-0.483	-0.381	0.241
MAT	-0.187	0.062	-0.288	-0.190	0.005
