# Supplementary material for: Global and local drivers of Echinococcus multilocularis infection in the western Balkan region
Source: Sci Rep. 2023 Dec 1;13:21176. doi: 10.1038/s41598-023-46632-9 (PMC10692075; doi:10.1038/s41598-023-46632-9)
Supplement: Supplementary file 9 — Supplementary Table 3. [file 41598_2023_46632_MOESM9_ESM.pdf]

Global and local drivers of *Echinococcus multilocularis* infection in the western Balkan region

Sibusiso Moloi, Tamás Tari, Tibor Halász, Bence Gallai, Gábor Nagy, Ágnes Csivincsik

Table S3. Location of the studied UTM grid cells**.**

UTM_GRID	latitude*	longitude	number of hosts
xm71d2	46.130597	17.280326	3
xm73c4	46.355458	17.323248	2
xm80a2	46.085231	17.343892	5
xm81a1	46.197382	17.349564	2
xm81b2	46.129426	17.345775	4
xm81d1	46.151165	17.409486	2
xm82b1	46.241823	17.351142	2
xm82c3	46.284757	17.449853	2
xm82d4	46.217541	17.446445	3
xm83a1	46.376523	17.356098	2
xm83a2	46.354768	17.354387	2
xm83d4	46.308085	17.451164	3
xm91a2	46.171550	17.476595	3
xm91b3	46.149035	17.507817	4
xm92a1	46.284538	17.483479	2
xm92a2	46.261904	17.480545	4
xm92a4	46.261166	17.513438	2
xm92b4	46.215812	17.512859	3
xm95c4	46.529061	17.590123	3
xm97c3	46.732053	17.600761	3
yl17c1	45.827100	17.783100	6
yl17c3	45.824550	17.815893	3
yl17c4	45.802450	17.814579	4
yl27a1	45.825852	17.848744	30
yl27a2	45.800649	17.846053	17
yl27a4	45.802459	17.876957	4
ym00a1	46.102110	17.602880	4
ym01a1	46.192548	17.606969	2
ym01b1	46.146539	17.605943	4
ym02a1	46.281460	17.610678	4
ym02a3	46.280783	17.643675	12
ym02a4	46.257872	17.642646	4
ym02c2	46.256928	17.673887	4
ym03a2	46.349853	17.615964	5
ym03b1	46.326571	17.612941	3
ym03b3	46.325157	17.645349	19
ym03b4	46.302810	17.643527	2

ym03d1	46.324823	17.677911	9
ym03d2	46.303062	17.676334	7
ym03d4	46.301708	17.709049	2
ym04c2	46.436707	17.683959	4
ym04c4	46.435339	17.714746	2
ym04d1	46.414685	17.682290	3
ym05a1	46.551538	17.623749	2
ym06c2	46.617524	17.692199	2
ym07a3	46.730747	17.665361	2
ym07d4	46.661679	17.726490	3
ym08b4	46.751415	17.667551	2
ym11a1	46.188165	17.736304	2
ym11a3	46.186749	17.769539	9
ym12a1	46.278195	17.740529	4
ym12a2	46.256407	17.739474	5
ym12a3	46.277965	17.772561	4
ym12a4	46.254705	17.772037	3
ym12b3	46.232670	17.771289	3
ym12c1	46.276883	17.806004	5
ym12c2	46.254431	17.804759	3
ym12c4	46.253506	17.836846	3
ym12d2	46.209181	17.802748	7
ym12d3	46.231101	17.835113	4
ym12d4	46.208230	17.834751	5
ym13b2	46.299240	17.740586	2
ym13b3	46.321723	17.776977	2
ym13c3	46.365553	17.842414	5
ym13c4	46.343575	17.840910	2
ym13d2	46.299083	17.806742	5
ym13d3	46.320825	17.841428	2
ym14d3	46.410685	17.844369	9
ym14d4	46.388617	17.842796	2
ym17b2	46.659971	17.760623	3
ym17c1	46.725602	17.827682	4
ym17c2	46.704145	17.827455	2
ym17c3	46.726067	17.860498	12
ym17c4	46.703658	17.860105	4
ym18c4	46.793122	17.863664	3
ym18d3	46.770939	17.862273	4
ym23a1	46.365692	17.874263	4
ym23a2	46.342135	17.873422	2
ym24b2	46.387355	17.876209	7
ym28a1	46.813893	17.899678	3
ym28a3	46.813835	17.931235	3
ym28b1	46.768737	17.895286	4
ym28b2	46.746920	17.895041	2
ym28c1	46.813325	17.964337	2
ym28c2	46.790003	17.963437	6
ym29b3	46.858749	17.934094	12

ym29b4	46.835194	17.932656	7

* Latitude and longitude indicate of the central coordinate of UTM quadrate.
