# Supplementary material for: Global and local drivers of Echinococcus multilocularis infection in the western Balkan region
Source: Sci Rep. 2023 Dec 1;13:21176. doi: 10.1038/s41598-023-46632-9 (PMC10692075; doi:10.1038/s41598-023-46632-9)
Supplement: Supplementary file 10 — Supplementary Legends. [file 41598_2023_46632_MOESM10_ESM.docx]

**Global and local drivers of Echinococcus multilocularis infection in the western Balkan region**

Sibusiso Moloi, Tamás Tari, Tibor Halász, Bence Gallai, Gábor Nagy, Ágnes Csivincsik

**Supplementary Information**

*Supplementary Table S1.* Hunting bag statistics of the studied areas (Baranya County and Somogy County) and Hungary.

*Supplementary Table S2.* Detailed results of the global (OLS) and local (GWR and MGWR) models.

*Supplementary Table S3.* Location of the studied UTM grid cells.

*Supplementary Figure S1.* Spatial distribution of mean coefficients of wetland (WET). (Used software for creation: QGIS, Białowieża version 3.22.)

*Supplementary Figure S2.* Spatial distribution of mean coefficients of mean annual precipitation (MAP). (Used software for creation: QGIS, Białowieża version 3.22.)

*Supplementary Figure S3.* Spatial distribution of mean coefficients of precipitation precipitation seasonality (PPT_SY). (Used software for creation: QGIS, Białowieża version 3.22.)

*Supplementary Figure S4.* Spatial variation of p-values of wetland (WET) obtained from a multiscale geographically weighted regression model. (Used software for creation: QGIS, Białowieża version 3.22.)

*Supplementary Figure S5.* Spatial variation of *p*-values of mean annual precipitation (MAP) obtained from a multiscale geographically weighted regression model. (Used software for creation: QGIS, Białowieża version 3.22.)

*Supplementary Figure S6.* Spatial variation of *p*-values of precipitation seasonality (PPT_SY) obtained from a multiscale geographically weighted regression model. (Used software for creation: QGIS, Białowieża version 3.22.)
